# The ethical imperative to treat NCDs during research in Africa

**DOI:** 10.1016/S2214-109X(19)30066-X

**Published:** 2019-04

**Authors:** Majaliwa Mzombwe, Bernard Desderius, Saidi Kapiga, Luke Smart, Robert Peck

**Affiliations:** Vanderbilt University School of Medicine, Nashville, TN 37232, USA; Department of Medicine, Catholic University of Health and Allied Sciences, Mwanza, Tanzania; London School of Hygiene and Tropical Medicine, Medical Research Council Tropical Epidemiology Group, London, UK; Mwanza Intervention Trials Unit, National Institute for Medical Research, Mwanza, Tanzania; Cincinnati Children’s Hospital Medical Center, Cincinnati, OH, USA; Center for Global Health and Department of Medicine, Division of General Internal Medicine, Weill Cornell Medical College, New York, NY, USA; Department of Medicine, Weill Bugando School of Medicine, Mwanza, Tanzania

As the rising prevalence and life-threatening consequences of non-communicable diseases (NCDs) in Africa have become more obvious,^1^ so too has the fact that many African health systems are ill-equipped to meet the health-care needs of local communities affected by NCDs.^2^ Meanwhile, the quantity of clinical research done in Africa continues to increase,^3^ and carries with it unique ethical questions, especially relating to the amount of NCD medical care that ought to be provided for participants involved in this research. Many African research participants, whether enrolled in studies that investigate NCDs or other diseases, will inevitably have NCDs, but have little to no access to NCD health care in their communities. NCDs present a unique challenge because, contrary to acute infectious processes, the treatment of NCDs is not usually urgent and a lack of care will not lead to immediate morbidity or mortality. As such, do researchers in Africa have a responsibility to provide ancillary NCD services to research participants? In light of the third UN High-Level Meeting on NCDs, our aim is to provoke a dialogue regarding ancillary NCD services in the context of research in Africa. We believe that this dialogue can be guided by several lessons learned during the HIV epidemic and by a moral responsibility to provide ancillary care as articulated by Richardson.^4^

First, researchers in Africa should strive for the “highest achievable standard” of ancillary care for NCDs diagnosed during the course of medical research.^5^ Setting standards that are too high for ancillary NCD services will make the conduct of research too burdensome.^6,7^ By contrast, accepting health inequity as an unchangeable reality misses the opportunity for research to combat these inequities.^5,8–11^ Determining this “highest achievable standard” will require an in-depth understanding of the social, economic, and political contexts of the settings for each study.^5^

Second, the “highest achievable standard” should be continuously reassessed and increased on the basis of improvements in the local health-care system and new scientific knowledge. During the initial decades (1995–2010) of HIV research in Africa, for example, the local capacity to provide antiretroviral treatment (ART) increased dramatically.^12^ Therefore, the standard for ancillary HIV care in Africa also increased to the point of ART being provided for participants in HIV trials.^8,12,13^ Members of one HIV trial network noted that, “One research organization cannot reverse global inequities in HIV[−1] care but researchers…have an obligation to try to narrow the equity gap.”^12^

Third, study-specific guidelines regarding ancillary care for NCDs should be guided by the “four Ps”: positive duty, planning, partnership, and practical provisions.^10^ Researchers have a duty to assist study participants diagnosed with NCDs: careful planning is needed to anticipate the NCD burden in the study population; NCD care plans should be developed in partnership with the local community to prevent displacing or disrupting the local health-care system; and investigators should clearly state the planned NCD provisions.

Fourth, study-specific ancillary care plans for NCDs should be guided by the nature of the relationships between the study participants, the investigators, and the community.^4^ One working group suggested a series of questions helpful in evaluating ancillary care needs, including “how severe or acute are the likely ancillary care needs”, is the identification of NCDs “integral or incidental to carrying out study procedures”, and “how extensive are the proposed interactions between researchers and study participants”.^10^ The final plan for ancillary NCD services should be the result of a moral negotiation between community members and the research study team.^11^

Finally, in our own research^14,15^ in east Africa, we have learned that high-quality ancillary services for common NCDs can be provided through the local health system at a reasonable cost. For example, in a cohort study of adults with HIV funded by the US National Institutes of Health, we raised modest private funds to provide free treatment for hypertension and diabetes. The HIV clinic used these funds to purchase high-quality generic drugs from a local pharmacy. Furthermore, we helped the HIV clinic’s case manager to develop a system to track blood pressure, blood glucose control, and prescription refills. We also worked with HIV staff to incorporate hypertension and diabetes screening into routine clinic services and to develop locally relevant NCD health education.

In conclusion, the medical research community in Africa has the opportunity and the moral responsibility to help combat the NCD epidemic in Africa through the provision of appropriate ancillary services for NCDs. Further dialogue is needed to help researchers and sponsors articulate the extent of this moral imperative.
